# Supplementary material for: A causal learning framework for the analysis and interpretation of COVID-19 clinical data
Source: PLoS One. 2022 May 19;17(5):e0268327. doi: 10.1371/journal.pone.0268327 (PMC9119448; doi:10.1371/journal.pone.0268327)
Supplement: S1 Table — Parameters used for the BSL and BDT analyses. (PDF) [file pone.0268327.s001.pdf]

Algorithm	Parameter	Value
BSL analyses	Significance level for retaining one edge	0.05
	Maximum size of the conditioning sets	Infinite
	Conditional independence test (cont. variables)	Fisher's z-transformation
	Conditional independence test (binary variables)	G square test
Binary decision tree	Max depth	4
	Min samples for split	2
	Measure of the quality of a split	Gini impurity
