# Supplementary material for: A causal learning framework for the analysis and interpretation of COVID-19 clinical data
Source: PLoS One. 2022 May 19;17(5):e0268327. doi: 10.1371/journal.pone.0268327 (PMC9119448; doi:10.1371/journal.pone.0268327)
Supplement: S2 Table — Results obtained with the SVM (linear and polynomial kernels) and Logistic Regression algorithms. In all the 3 cases, the permutation test using random features shows significantly inferior results with respect to the features chosen with the causal analysis. Note that the results of the permutation tests are the average of those obtained from 1,000 permutations. (PDF) [file pone.0268327.s002.pdf]

Algorithm	Input data	Sensitivity		Specificity		F1 score	
		Train all	10f cv	Train all	10f cv	Train all	10f cv
SVM pol.	7 feat of the tree in Fig. 4	0.78	0.43	1.00	0.97	0.88	0.53
	7 random feat	0.19	0.14	0.98	0.96	0.28	0.19
SVM lin.	7 feat of the tree in Fig. 4	0.57	0.50	0.97	0.95	0.68	0.58
	7 random feat	0.19	0.16	0.97	0.96	0.27	0.21
LR	7 feat of the tree in Fig. 4	0.67	0.62	0.95	0.94	0.73	0.66
	7 random feat	0.31	0.27	0.95	0.94	0.40	0.33
