# Supplementary figures and images for: A causal learning framework for the analysis and interpretation of COVID-19 clinical data
Source: PLoS One. 2022 May 19;17(5):e0268327. doi: 10.1371/journal.pone.0268327 (PMC9119448; doi:10.1371/journal.pone.0268327)

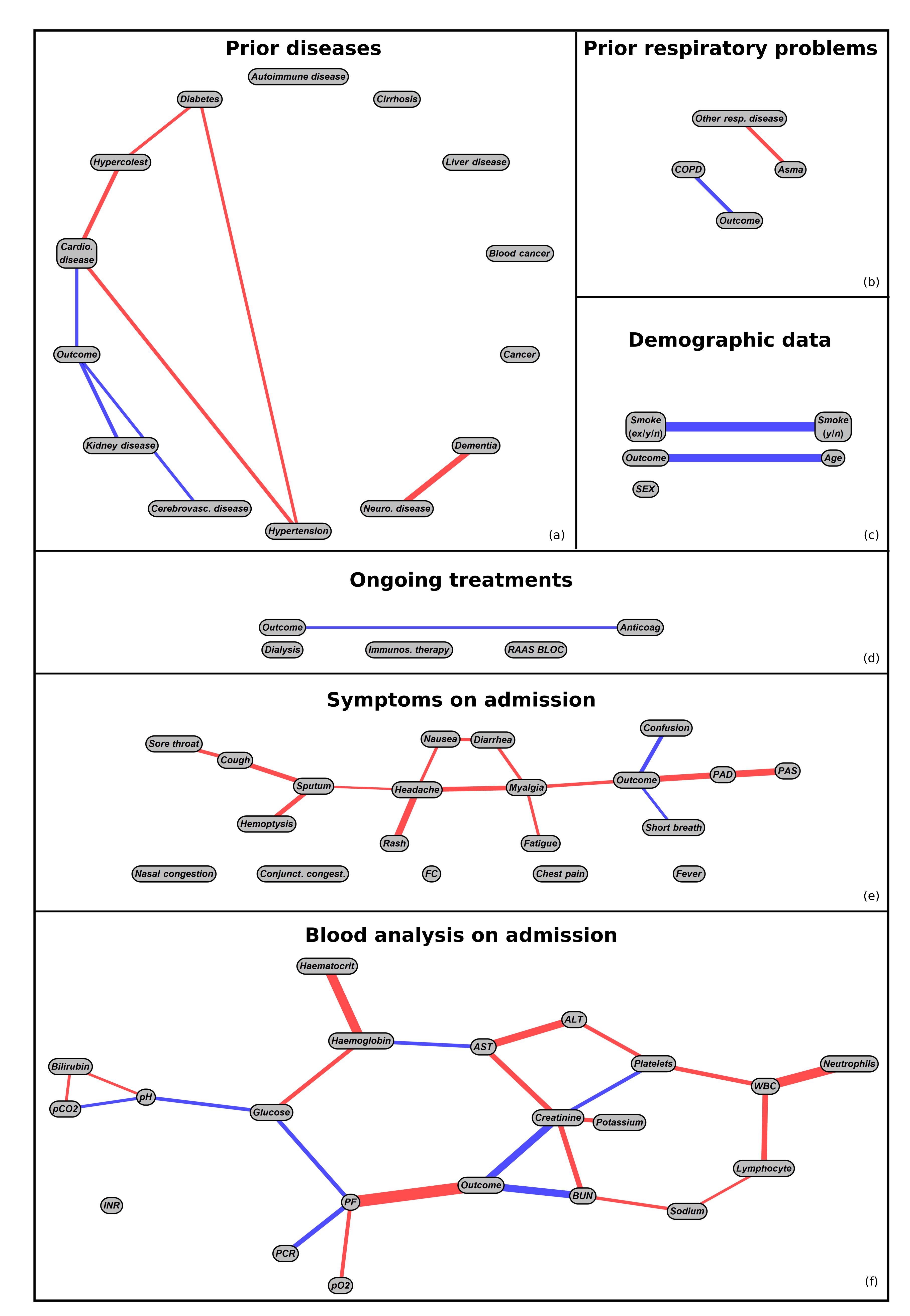

Supplement: S1 Fig — Image showing the BSL analysis applied to different categories of features. All the illustrated graphs are generated without taking the information provided by clinicians into account. (TIF) [file pone.0268327.s003.tif]

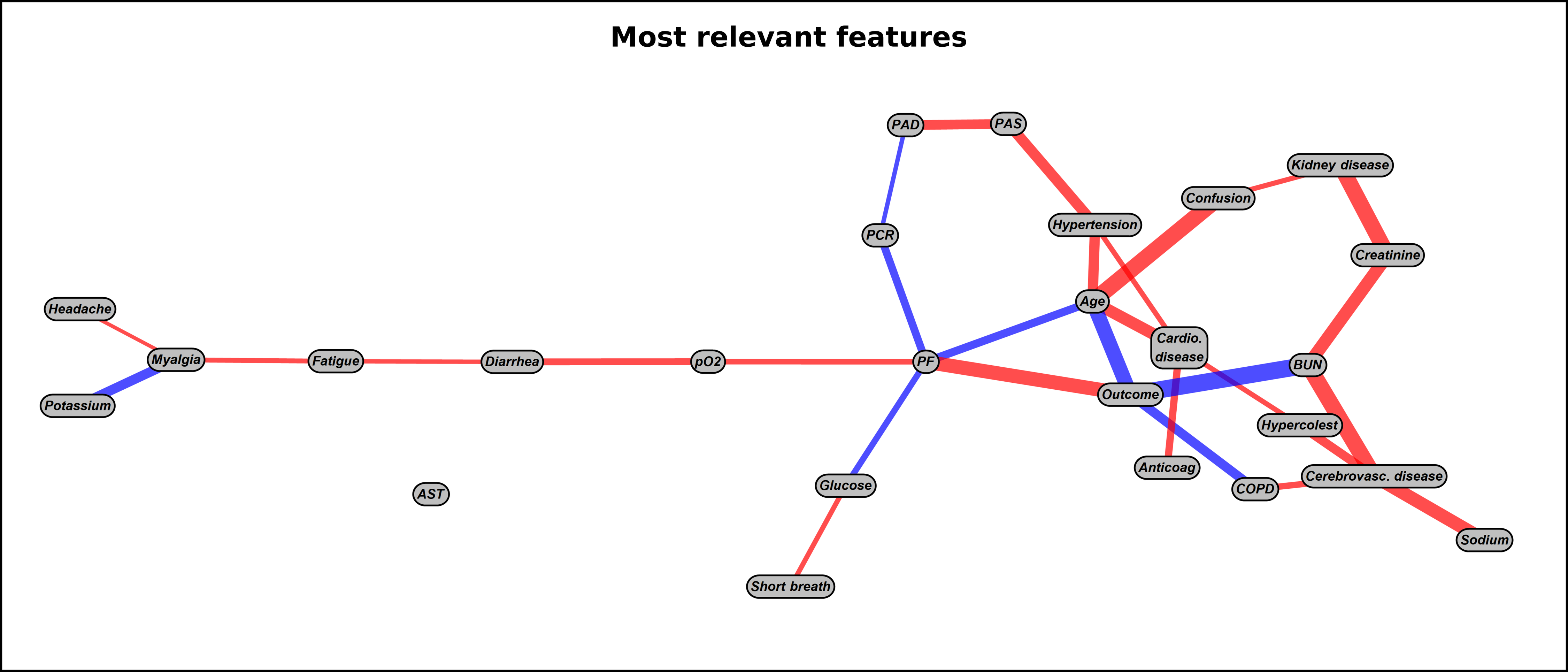

Supplement: S2 Fig — Graph generated with the most relevant features found from the graphs shown in S1 Fig. This graph is generated without taking the information provided by clinicians into account. (TIF) [file pone.0268327.s004.tif]

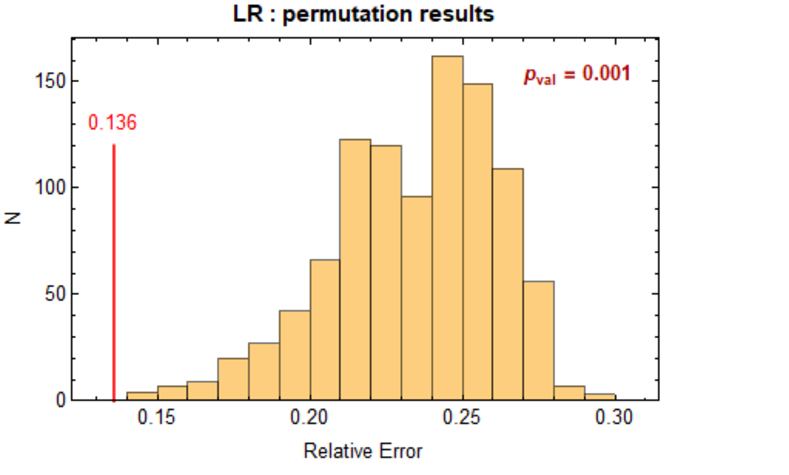

Supplement: S3 Fig — Results of the permutation test conducted with the logistic regression algorithm. (TIF) [file pone.0268327.s005.tif]

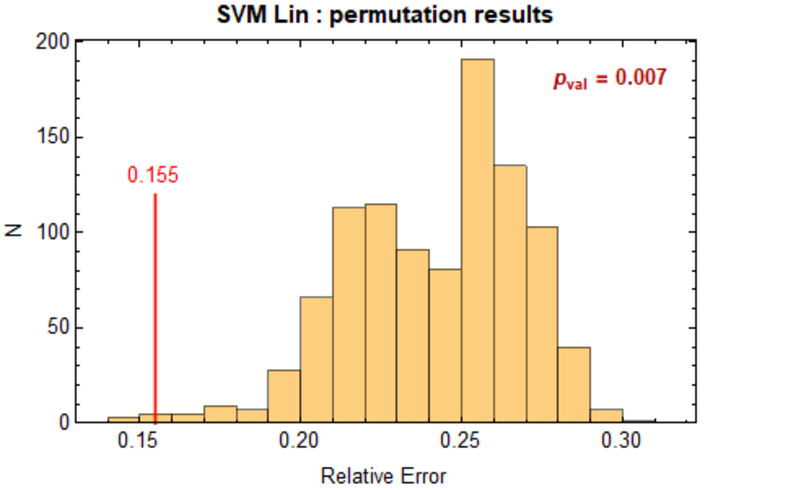

Supplement: S4 Fig — Results of the permutation test conducted with the linear kernel SVM. (TIF) [file pone.0268327.s006.tif]

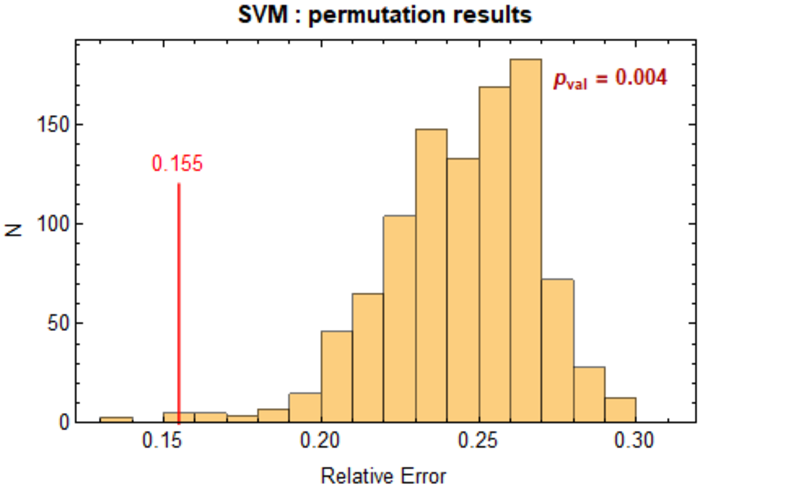

Supplement: S5 Fig — Results of the permutation test conducted with the polynomial kernel SVM. (TIF) [file pone.0268327.s007.tif]
